# The Role of Transient Receptor Potential Channel 6 Channels in the Pulmonary Vasculature

**DOI:** 10.3389/fimmu.2017.00707

**Published:** 2017-06-16

**Authors:** Monika Malczyk, Alexandra Erb, Christine Veith, Hossein Ardeschir Ghofrani, Ralph T. Schermuly, Thomas Gudermann, Alexander Dietrich, Norbert Weissmann, Akylbek Sydykov

**Affiliations:** ^1^Excellence Cluster Cardio-Pulmonary System, Universities of Giessen and Marburg Lung Center (UGMLC), German Center for Lung Research (DZL), Justus Liebig University of Giessen, Giessen, Germany; ^2^Walther Straub Institute for Pharmacology and Toxicology, Ludwig Maximilian University of Munich, German Center for Lung Research (DZL), Munich, Germany

**Keywords:** transient receptor potential channels, transient receptor potential channel 6, hypoxic pulmonary vasoconstriction, pulmonary hypertension, vascular permeability

## Abstract

Canonical or classical transient receptor potential channel 6 (TRPC6) is a Ca^2+^-permeable non-selective cation channel that is widely expressed in the heart, lung, and vascular tissues. The use of TRPC6-deficient (“knockout”) mice has provided important insights into the role of TRPC6 in normal physiology and disease states of the pulmonary vasculature. Evidence indicates that TRPC6 is a key regulator of acute hypoxic pulmonary vasoconstriction. Moreover, several studies implicated TRPC6 in the pathogenesis of pulmonary hypertension. Furthermore, a unique genetic variation in the TRPC6 gene promoter has been identified, which might link the inflammatory response to the upregulation of TRPC6 expression and ultimate development of pulmonary vascular abnormalities in idiopathic pulmonary arterial hypertension. Additionally, TRPC6 is critically involved in the regulation of pulmonary vascular permeability and lung edema formation during endotoxin or ischemia/reperfusion-induced acute lung injury. In this review, we will summarize latest findings on the role of TRPC6 in the pulmonary vasculature.

## Introduction

Regulation of the intracellular Ca^2+^ ([Ca^2+^]_i_) homeostasis is a crucial factor in many physiological processes (1). Altered Ca^2+^ homeostasis in both vascular endothelium and smooth muscle has been documented for a majority of pathophysiological conditions in the pulmonary vasculature. Changes in [Ca^2+^]_i_ play a pivotal role in the regulation of contraction, migration, and proliferation of vascular smooth muscle cells (2). Furthermore, Ca^2+^ signaling in endothelial cells (ECs) is essential for the maintenance of the endothelial barrier integrity (3).

Non-selective cation channels (NSCCs) play an important role in the regulation of vascular tone and vascular smooth muscle cell proliferation by mediating the entry of cations (4). Among the ion channels located in the pulmonary vasculature, members of the canonical or classical transient receptor potential (TRPC) channels subfamily allow for the entry of Na^+^ and Ca^2+^. There is growing evidence that transient receptor potential channel 6 (TRPC6) mediates receptor-operated cation entry and is critically involved in numerous physiological processes. Recent studies have provided important insights into the role of TRPC6 in normal physiology and disease states of the pulmonary vasculature. We provide an overview on current knowledge regarding the role of TRPC6 channels in pulmonary vasculature and potential therapeutic strategies.

## Regulation of Calcium Homeostasis

**Figure 1:**
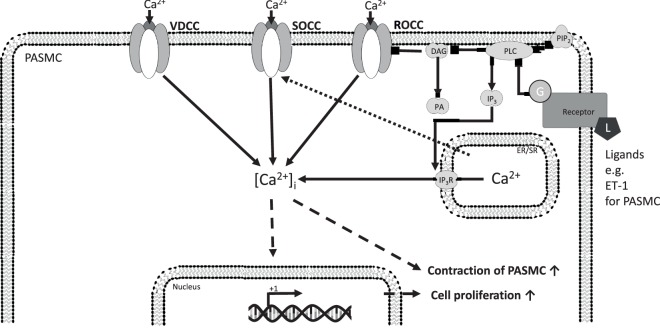
[Ca^2+^]_i_ homeostasis regulation in precapillary pulmonary arterial smooth muscle cells (PASMCs) and ECs. Ca^2+^ enters cells from extracellular fluid through L-type voltage-dependent calcium channels or non-selective cation channels, which can be divided into SOCCs and ROCCs. The initiation of ROCC-mediated Ca^2+^-influx from the extracellular space is thought to be induced by ligand-activated G-protein coupled receptors, starting a PLC-mediated hydrolyzation of PIP_2_ to IP_3_ and DAG. DAG regulates the activity of ROCC to induce receptor-operated Ca^2+^ entry, whereas IP_3_ generation induces depletion of the intracellular Ca^2+^ stores in the endoplasmic reticulum, leading to induction of store-operated Ca^2+^ entry. The increased [Ca^2+^]_i_ drives different cellular responses. Ca^2+^, calcium ion; [Ca^2+^]_i_, intracellular Ca^2+^ concentration; ROCC, receptor-operated calcium channel; SOCC, store-operated calcium channel; VDCC, L-type voltage-dependent calcium channel; DAG, diacylglycerol; DAGK, DAG kinase; EC, endothelial cell; ER/SR, endoplasmic/sarcoplasmic reticulum; IP_3_, inositol trisphosphate; IP_3_R, inositol trisphosphate receptor; L, ligand; PA, phosphatidic acid; PASMC, precapillary pulmonary arterial smooth muscle cells; PIP_2_, phosphatidylinositol 4,5-bisphosphate; PLC, phospholipase C; VEGF, vascular endothelial growth factor; solid arrows indicate direct interactions; dotted arrows illustrate indirect interactions.

In general, Ca^2+^ enters cells from extracellular fluid through L-type voltage-dependent calcium channels or NSCCs, which can be divided into store-operated calcium channels (SOCCs) and receptor-operated calcium channels (ROCCs) (Figure 1). Stimulation of G-protein-coupled receptors initiates signaling mechanisms leading to activation of ROCC. These signaling pathways include phospholipase C (PLC) activation resulting in production of diacylglycerol (DAG) along with inositol 1,4,5-trisphosphate (IP_3_) from phosphatidylinositol 4,5-bisphosphate (PIP_2_). DAG regulates the activity of ROCC to induce receptor-operated Ca^2+^ entry, whereas IP_3_ generation induces depletion of the intracellular Ca^2+^ stores in the endoplasmic reticulum, leading to induction of store-operated Ca^2+^ entry. Ca^2+^ entry through SOCCs plays a very important role in Ca^2+^ stores replenishment in the endoplasmic/sarcoplasmatic reticulum and maintaining Ca^2+^ homeostasis.

## Classical Transient Receptor Potential Channel 6

Transient receptor potential (TRP) channels play a prominent role in the regulation of the cation homeostasis (5). TRP channels belong to a large and diverse family of mostly NSCCs. In this regard, they are non-selectively permeable to cations, including potassium (K^+^), sodium (Na^+^), calcium (Ca^2+^), and magnesium (Mg^2+^) (6). Based on amino acid sequence homology, the 28 mammalian TRP channels are grouped into six subfamilies, one of which is the TRPC (for classical or canonical) subfamily (6). The TRPC subfamily includes seven members, TRPC1 to TRPC7, and can be further divided into subfamilies on the basis of their structural and functional similarities. All TRPC proteins have a common structure. Mainly, they are composed of four N-terminal ankyrin repeats, six transmembrane domains with a putative pore between domains 5 and 6, and several protein-binding domains (4). TRPC proteins can form homomeric or heteromeric channels consisting of four monomers.

Transient receptor potential channel 6 is a NSCC, which is about six times more permeable for Ca^2+^ than for Na^+^ (7). It belongs to the subfamily of ROCC and there is good evidence that TRPC6 is directly activated by DAG (8). TRPC6 is ubiquitously expressed in the whole vasculature (9). In the pulmonary circulation, TRPC6 is most prominent in pulmonary artery smooth muscle cells (PASMCs) and ECs (10). TRPC6 mRNA and protein were identified in PASMCs isolated from both proximal and distal pulmonary arteries (11–13). However, TRPC6 expression is higher in PASMCs isolated from distal pulmonary arteries than in those isolated from proximal vessels (14). Recently, expression of TRPC6 in pulmonary venous smooth muscle cells has also been demonstrated (15).

## Pulmonary Hypertension

Pulmonary hypertension (PH) is a pathophysiological disorder that may involve various clinical conditions and can complicate cardiovascular and respiratory diseases (16). PH is characterized by remodeling of the pulmonary vessels, leading to a progressive increase in pulmonary vascular resistance (PVR), right ventricular failure, and premature death. PH is defined as a resting mean pulmonary artery pressure ≥25 mmHg (17). The disorder has been classified into five clinical groups based on their similarities in clinical presentation, pathophysiological mechanisms, and therapeutic options: pulmonary arterial hypertension (PAH) (Group 1); PH due to left heart disease (Group 2); PH due to chronic lung disease and/or hypoxia (Group 3); chronic thromboembolic PH (Group 4); and PH due to unclear and/or multifactorial mechanisms (Group 5) (18).

Depending on the pulmonary artery wedge pressure values, PH is divided into precapillary and postcapillary forms. Pulmonary artery wedge pressure provides an indirect estimate of left atrial pressure and its elevation >15 mmHg in patients with PH indicates presence of postcapillary PH due to left heart disease (Group 2) (17). Precapillary PH is defined by the presence of PH and a pulmonary artery wedge pressure ≤15 mmHg and includes the clinical groups 1, 3, 4, and 5 (17).

Pulmonary arterial hypertension is a progressive disease characterized by the presence of precapillary PH and a PVR > 3 Wood units in the absence of other causes of precapillary PH (17). It includes idiopathic PAH (IPAH), hereditary PAH, and PAH associated with diseases, drugs, and toxins (APAH) (18). Sustained pulmonary vasoconstriction, *in situ* thrombosis, and pathological pulmonary vascular remodeling due to excessive vascular cell growth leading to intimal narrowing and vascular occlusion are the main causes for the increased PVR and pulmonary arterial pressure in IPAH patients. In addition, pulmonary vascular remodeling with increased muscularization contributes to elevated PVR as well as hyperreactivity of pulmonary vessels to various vasoconstrictor agents. Neointimal and medial hypertrophy in small and medium-sized pulmonary arteries is a key aspect of pulmonary vascular remodeling in IPAH patients.

### Role of TRPC6 in Hypoxic Pulmonary Vasoconstriction (HPV)

Acute HPV is an adaptive response of the pulmonary circulation to a local alveolar hypoxia, by which local lung perfusion is matched to ventilation resulting in optimization of ventilation–perfusion ratio and thus gas exchange (19, 20). This dynamic mechanism is also known as von Euler–Liljestrand mechanism (21) and can be found in fish, reptiles, birds, and mammals. Acute HPV occurs throughout the pulmonary vascular bed, including arterioles, capillaries, and veins, but is most pronounced in small pulmonary arterioles (22, 23). In isolated pulmonary arteries and isolated perfused lungs, the HPV response is typically biphasic (24–26). The first phase is characterized by a fast but mostly transient vasoconstrictor response that starts within seconds and reaches a maximum within minutes. The following second phase is characterized by a sustained pulmonary vasoconstriction. Acute HPV in local alveolar hypoxia is limited to the affected lung segments and is not accompanied by an increase in pulmonary artery pressure.

**Figure 2:**
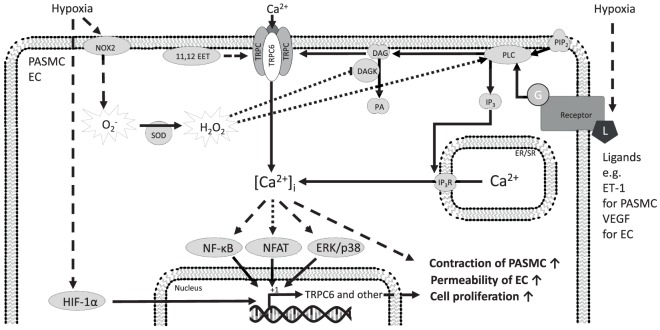
Mechanisms of TRPC6 regulation and function in precapillary pulmonary arterial smooth muscle cells (PASMCs) and ECs in response to hypoxia. The TRPC6 protein forms homomeric and heteromeric channels composed of TRPC6 alone or TRPC6 and other TRPC proteins. TRPC6 is expressed in PASMCs from mice, rat, as well as humans and is suggested to play a significant role in human idiopathic PAH. The initiation of TRPC6-mediated Ca^2+^ influx from the extracellular space is thought to be induced by ligand-activated G-protein coupled receptors, starting a PLC-mediated hydrolyzation of PIP_2_ to IP_3_ and DAG. It has been already shown that DAG activates TRPC6-containing channels to induce Ca^2+^ influx from the extracellular space. Ca^2+^ entry through TRPC6 might be triggered by hypoxia-induced ${\text{O}}_{2}^{-}$ production or hypoxia-induced DAG accumulation and that the increased [Ca^2+^]_i_ drives different cellular responses through ERK and p38, NFAT, and NF-κB downstream signaling. These pathways might be involved in the induction of TRPC6 expression and contribute to the modulated cellular response associated with hypoxia. Moreover, hypoxia leads to acute stabilization of HIF-1α, which might induce TRPC6 expression among other proteins. 11,12 EET, 11,12-epoxyeicosatrienoic acid; Ca^2+^, calcium ion; [Ca^2+^]_i_, intracellular Ca^2+^ concentration; DAG, diacylglycerol; DAGK, DAG kinase; EC, endothelial cell; ER/SR, endoplasmic/sarcoplasmic reticulum; ERK, extracellular signal-regulated kinase; ET-1, endothelin-1; G, G-protein; H_2_O_2_, hydrogen peroxide; HIF-1α, hypoxia-inducible factor 1 alpha; IP_3_, inositol trisphosphate; IP_3_R, inositol trisphosphate receptor; L, ligand; NF-κB, nuclear factor kappa-light-chain enhancer of activated B-cells; NFAT, nuclear factor of activated T-cells; NOX2, NADPH (nicotinamide adenine dinucleotide phosphate) oxidase 2; ${\text{O}}_{2}^{-}$, superoxide; PA, phosphatidic acid; p38, p38 mitogen-activated protein kinase; PASMC, precapillary pulmonary arterial smooth muscle cells; PIP_2_, phosphatidylinositol 4,5-bisphosphate; PLC, phospholipase C; SOD, superoxide dismutase; TRPC, classical transient receptor potential channel; TRPC6, classical transient receptor potential channel 6; VEGF, vascular endothelial growth factor; solid arrows indicate direct interactions; dotted arrows illustrate indirect interactions. Not all interaction partners have been identified.

A rise of [Ca^2+^]_i_ in PASMCs is a key element in HPV (27, 28). We have demonstrated that TRPC6 plays an essential role in acute HPV (29). We have shown that the first acute phase of HPV (<20 min of hypoxic exposure) was completely abolished in isolated, ventilated, and buffer-perfused lungs from TRPC6-deficient mice. However, the vasoconstrictor response during the second sustained phase (60–160 min of hypoxic exposure) in TRPC6^−/−^ mice was not significantly different from that in wild-type mice (29). During hypoxia, DAG is accumulated in PASMCs and leads to activation of TRPC6 (29). Accumulation of DAG can result from PLC activation or from ROS-mediated DAG kinase (DAGK) inhibition (30, 31). Along these lines, inhibition of DAG synthesis by the PLC inhibitor U73122 inhibited acute HPV in wild-type mouse lungs (32). Blocking DAG degradation to phosphatidic acid through DAGKs or activation of TRPC6 with a membrane-permeable DAG analog 1-oleoyl-2-acetyl-sn-glycerol (OAG) resulted in normoxic vasoconstriction in wild-type but not in TRPC6^−/−^ mice (32). Recently, the cystic fibrosis transmembrane conductance regulator and sphingolipids have been demonstrated to regulate TRPC6 activity in HPV, as both translocate TRPC6 channels to the caveolae and activate the PLC–DAG–TRPC6 pathway (33). Cytochrome P-450 epoxygenase-derived epoxyeicosatrienoic acids also induced translocation of TRPC6 to the caveolae during acute hypoxia (34). Consistent with these data, 11,12-epoxyeicosatrienoic acids increased pulmonary artery pressure in a concentration-dependent manner and potentiated HPV in heterozygous but not in TRPC6-deficient lungs (34). As the constriction of the pulmonary vessels in response to the thromboxane mimetic U46619 is not altered in TRPC6^−/−^ mice, TRPC6 channels appear to be a key regulator of acute HPV. These studies are summarized in Figure 2.

In PASMCs isolated from small precapillary arteries of TRPC6-deficient mice, cation influx and currents induced by severe hypoxia (1% O_2_) were completely absent (29). The rise of [Ca^2+^]_i_ in response to hypoxia was not dependent on Ca^2+^ release from internal stores, because, in the absence of extracellular Ca^2+^, no hypoxia-induced increases in [Ca^2+^]_i_ were detected (29). Interestingly, blocking voltage-gated Ca^2+^ channels almost completely inhibited acute HPV in isolated wild-type mouse lungs and Ca^2+^ influx in wild-type PASMCs (29), suggesting that Na^+^ influx through TRPC6 channels leads to membrane depolarization and activation of voltage-gated L-type Ca^2+^ channels mediating the bulk of the Ca^2+^ influx and contraction of smooth muscle cells (35). Importantly, the lack of acute HPV in TRPC6 KO mice has profound physiological relevance, because partial occlusion of alveolar ventilation provoked severe hypoxemia in TRPC6^−/−^ but not in wild-type mice (29). These data provide compelling evidence that different molecular mechanisms regulate pulmonary vascular responses to acute and sustained hypoxia. TRPC6 channels may thus represent a potential therapeutic target for the control of pulmonary hemodynamics and gas exchange in hypoxic conditions.

### Role of TRPC6 in Experimental PH

A variety of animal models are currently used to study PH. These models have provided a plethora of scientific information and made significant contribution to our understanding of molecular mechanisms in PH. In animals, PH can be induced by pharmacologic/toxic substances, genetic manipulations, exposure to environmental factors, or surgical interventions (36).

Exposure to chronic hypoxia is the most commonly used animal model of PH in biomedical research. In global alveolar hypoxia, which occurs at high altitude and chronic respiratory diseases, HPV involves the entire pulmonary vascular bed leading to elevation of pulmonary artery pressure. Chronic global alveolar hypoxia induces structural remodeling of pulmonary vessels due to smooth muscle cell proliferation and migration characterized by increased muscularization of smaller arteries with extension of smooth muscle cells into previously non-muscularized arterioles (37). This vascular remodeling has previously been thought to be a major determinant of the persistent elevation of PVR in chronic hypoxia-induced PH (38–40). However, recent studies have provided evidence that sustained vasoconstriction is an important contributor to chronic hypoxia-induced PH (41).

Although TRPC6 is important in the acute phase of HPV in mouse lungs, the data regarding its role in chronic hypoxic PH are controversial. We have previously shown that, despite disrupted acute HPV, TRPC6-deficient mice display sustained HPV and chronic hypoxia-induced PH with pulmonary vascular remodeling and RV hypertrophy after 3 weeks of hypoxia (10% O_2_), which are indistinguishable from those in wild-type mice (29). Slightly but significantly lower right ventricular systolic pressure was observed in TRPC6^−/−^ mice exposed to 1 week of hypoxia when compared to wild-type mice (42). Nevertheless, this difference was not significant after 3 weeks of exposure to hypoxia (42). In contrast, other authors have demonstrated attenuation of PH and pulmonary vascular remodeling in TRPC6 KO mice after 4 weeks of hypoxia (43). Although the exact reason is not clear, differences in age (44, 45), gender (46), strain, and substrain (47, 48) of mice can account for most of the discrepancies.

Excessive proliferation of PASMCs is the main cause of pulmonary arterial medial hypertrophy, which narrows the intraluminal diameter, increases the resistance to blood flow, and eventually leads to PH. Proliferation of PASMCs is regulated by [Ca^2+^]_i_. There is increasing evidence that elevated TRPC6 expression might be responsible for the elevated [Ca^2+^]_i_. Interestingly, it has been shown that enhanced expression of TRPC6, STIM2, and Orai2 as proteins of the store-operated Ca^2+^ influx underlies the change of the phenotype of PASMCs from the contractile to the proliferative (49). Furthermore, deletion of TRPC6 significantly attenuated Ca^2+^ currents in the proliferative phenotype of PASMCs (49).

Transient receptor potential channel 6 upregulation in PASMCs has been demonstrated to be dependent on hypoxia-inducible transcription factor 1 (HIF-1) (50). Overexpression of HIF-1 led to TRPC6 upregulation under normoxic conditions while partial deficiency in HIF-1 resulted in hypoxia-induced Ca^2+^ influx in PASMCs, suggesting an important role of HIF-1 for sustained expression of TRPC6 channels (50). Although short-term hypoxia (1% O_2_ for 72 h) did not produce any changes in TRPC6 mRNA expression in isolated murine PASMCs (51), increased expression of TRPC6 on mRNA and protein level was detected in pulmonary arteries and PASMCs isolated from pulmonary arteries of mice and rats exposed to chronic hypoxia (42, 50, 52). Moreover, a Notch-dependent upregulation of TRPC6 channels in PASMCs in response to chronic hypoxia has recently been reported (43). It has been shown that TRPC6 is induced by BMP4 in rat PASMCs *via* the p38MAPK and ERK1/2 pathways (53, 54). Additionally, BMP4 may increase TRPC6 expression by elevating NOX4-mediated ROS levels in PASMCs (55). Interestingly, BMP4 expression has been shown to be dependent on HIF-1 as well (15).

Chronic lung diseases including chronic obstructive pulmonary disease (COPD) are often complicated by PH (56). Growing evidence implicates cigarette smoke (CS) products in the initiation of pulmonary vascular alterations in COPD (57). Recently, we have demonstrated the formation of PH in mice chronically exposed to tobacco smoke (58). Similarly, development of PH has been documented in rats chronically exposed to CS (59, 60). CS is an inflammatory stimulus, which upregulates Ca^2+^-regulatory molecules. In this regard, TRPC6 was upregulated in rat lungs and isolated rat PASMCs after 4, 12, and 20 weeks of CS exposure (59). In another study, expression of TRPC1 and TRPC6 was increased in PASMCs isolated from distal pulmonary arteries of rats after 1, 3, and 6 months of CS exposure (60). Furthermore, PASMCs in rats exposed to CS for 3 and 6 months showed a higher basal [Ca^2+^]_i_ and an increased Ca^2+^ entry (60).

The role of TRPC6 in other models of PH has not been investigated in detail. Increased expression of TRPC6 protein in distal pulmonary arteries was observed in the monocrotaline-induced rat model of PH (61). Chronic thromboembolic PH in a rat model is associated with upregulation of TRPC1 and TRPC6 in PASMCs isolated from distal pulmonary arteries, elevated basal [Ca^2+^]_i_, and an increased Ca^2+^ entry (62).

There is growing evidence that in addition to TRPC6, other members of the TRPC family also contribute to the pulmonary vascular remodeling in PH. Culture of isolated PASMCs under hypoxic conditions led to upregulation of TRPC1 mRNA (50, 51, 63). Furthermore, enhanced expression of TRPC1 and TRPC4 mRNA and protein has been documented in pulmonary arteries and PASMCs isolated from mice and rats with PH induced by various stimuli (50, 59, 60, 62, 64, 65). Treatment of murine PASMCs with TRPC1-specific small interfering RNA resulted in significant attenuation of hypoxia-induced proliferation of cells (51). Consistent with this, PASMCs isolated from TRPC1^−/−^ mice showed diminished proliferation under hypoxic conditions (51). Additionally, TRPC1^−/−^ mice exposed to chronic hypoxia were protected from development of PH, which was associated with attenuated pulmonary vascular remodeling (51). In line with our data, reduced chronic hypoxic vascular remodeling in TRPC1^−/−^ mice has been demonstrated by an independent research group (42). Moreover, downregulation of TRPC1 expression by small interfering RNA attenuated PH and pulmonary vascular remodeling in a murine model of hypoxia-induced PH (66). Interestingly, in mice deficient for both TRPC1 and TRPC6, chronic hypoxia-induced changes in pulmonary arterial pressure, right ventricular hypertrophy, and pulmonary vascular remodeling are even more inhibited compared to those in mice with a deficiency for a single gene (42). In a recent study, deficiency for TRPC4 has been shown to confer a survival benefit, which was associated with diminished vasculopathy in a rat model of severe PAH (67).

### Involvement of TRPC6 in IPAH

Pulmonary arterial hypertension is characterized by progressive adverse structural changes in the resistance pulmonary arteries driven mainly by excessive vascular cell growth (68). Vascular remodeling in PAH is mediated by multiple stimuli. It is widely recognized that PASMCs in IPAH patients have a hyperproliferative phenotype and contribute to the pro-proliferative microenvironment in the vascular wall of their pulmonary arteries (68, 69). The enhanced [Ca^2+^]_i_ plays an key role in PASMC growth (70). Furthermore, increased [Ca^2+^]_i_ levels have been observed in PASMCs from IPAH patients (71). Expression studies revealed that c-jun/STAT3-induced upregulation of TRPC6 expression underlies PDGF-mediated proliferation of PASMCs (72). The mRNA and protein expression of TRPC6 in lung tissues and PASMCs from IPAH patients has been shown to be much higher than in those from normotensive patients (73). Furthermore, inhibition of TRPC6 gene expression by small interfering RNA significantly diminished proliferation of PASMCs from IPAH patients suggesting that the abnormally increased PASMC proliferation in these patients may be due to enhanced expression of TRPC6 (73).

Mounting evidence implicates inflammatory mechanisms in the development of PAH (74, 75). A unique genetic variant of the TRPC6 gene promoter has been identified, which might link inflammatory responses to the upregulation of TRPC6 expression and ultimate development of pulmonary vascular abnormality in IPAH (76). Sequencing TRPC6 regulatory regions of 268 patients with IPAH revealed three biallelic single-nucleotide polymorphisms (SNPs): −361(A>T), −254(C>G), and −218(C>T) (76). Among these three SNPs, only the −254(C>G) SNP was associated with IPAH by increasing basal TRPC6 gene promoter activity. Furthermore, the −254(C>G) SNP introduces a new binding site for the inflammatory transcription factor nuclear factor κB (NF-κB) in the promoter region of the TRPC6 gene and thus enhances NF-κB-mediated promoter activity and stimulates TRPC6 expression in PASMCs (76). In addition, this SNP has functional relevance as it also affects TRPC6 channel activity. In PASMCs from IPAH patients with the −254(C>G) SNP, TNF-α-induced activation of NF-κB significantly increased TRPC6 expression, elevated the resting [Ca^2+^]_i_, and enhanced OAG-induced Ca^2+^ influx (76). In contrast, inhibition of nuclear translocation of NF-κB by overexpression of an IκBα super-repressor significantly diminished TNF-α-mediated enhancement of TRPC6 expression, resting [Ca^2+^]_i_, and agonist-induced elevation of [Ca^2+^]_i_. The importance of NF-κB has been demonstrated in experimental models of PAH (77–79). More importantly, activation of NF-κB has recently been observed in the pulmonary vessels of patients with end-stage IPAH (80). Thus, in the presence of inflammatory triggers, individuals carrying the −254(C>G) SNP may have an increased risk of developing IPAH (81). Although the functional significance of the two other SNPs, −361(A>T) and −218(C>T), is not clear, it has been shown that patients with IPAH and APAH carrying all three SNPs develop a more severe disease (82).

### TRPC6 As a Therapeutic Target in PH

Transient receptor potential channel 6 is predominantly expressed in tissues harboring smooth muscle cells including the lungs (83, 84). However, TRPC6^−/−^ mice do not have any major pathological phenotypes probably because TRPC6 channels have little basal activity and modest importance under physiological conditions (85). Moreover, loss of TRPC6 is compensated by the activity of closely related TRPC3 channels in the systemic vasculature (86) and airway smooth muscle (87). Nevertheless, TRPC6 channels are specifically activated in various disease conditions suggesting their pathophysiological relevance and thus represent attractive therapeutic targets. Importantly, systemic application of TRPC inhibitors in mice was not associated with any serious side effects (88, 89).

A number of non-selective small molecule inhibitors of TRPC6 channel activities including 2-APB and SKF-96365 have become available during recent years (85, 90). Also, antagonists including synthetic gestagen norgestimate and compound 8009-5364 with IC50 values in a low micromolar range and with higher selectivity for TRPC6 have been identified (91, 92). As the members of the TRPC3/6/7 subfamily have very similar biochemical and biophysical properties, most of the TRPC6-selective blockers exhibit poor selectivity between the subfamily members (85, 90). A continuous search for selectively acting pharmacological TRPC6 has recently identified new highly potent TRPC6 inhibitors with subtype selectivity, SAR7334, and larixyl acetate (93, 94). Most importantly, these drugs effectively blocked acute HPV in isolated mouse lungs (92–94). However, the only inhibitor that has been tested in experimental PH is the non-specific TRPC blocker 2-APB, which prevented development of PH in mice exposed to chronic hypoxia (43).

Evidence supporting the role of TRPC6 in the pathogenesis of IPAH suggests that it might serve as a pharmacologic target. Although the selective TRPC6 inhibitors represent promising drug candidates for the treatment of PH, they have not yet been tested in experimental models of PH. It would be highly desirable to confirm the therapeutic efficacy and safety of the new potent and selective TRPC6 blockers in animal models of PH with the ultimate goal of development of new therapeutic strategies for patients with PH.

Recent studies suggest that specific drugs approved for PAH treatments can also target TRPC6 expression and activity. In a small number of PAH patients with a positive response to acute vasodilator testing, initial therapy includes high doses of calcium channel blockers. However, most of the PAH patients do not react to calcium channel blockers, and they are treated with drugs approved for PAH therapy. Currently, established clinical practice treatments of PAH target three signaling pathways that are involved in the pathogenesis of PH: endothelin, nitric oxide, and prostacyclin (95). These therapies include endothelin receptor antagonists, phosphodiesterase type 5 inhibitors, soluble guanylate cyclase stimulators, prostacyclin receptor agonists, and epoprostenol. Bosentan has been found to directly downregulate TRPC6 expression in addition to its well-known blockade of endothelin receptors (96).

In PASMCs from chronically hypoxic rats, the potent phosphodiesterase type 5 inhibitor sildenafil decreased acutely basal [Ca^2+^]_i_ (97). Chronic treatment of rats exposed to 10% O_2_ for 21 days with sildenafil showed a decreased right ventricular pressure and right ventricular hypertrophy, which is related to decreased TRPC6 mRNA and protein expression in pulmonary arteries (63). Furthermore, knockdown of TRPC6 gene by small interference RNA diminished the hypoxic increases of basal [Ca^2+^]_i_ and Ca^2+^ influx in PASMCs exposed to hypoxia for 60 h (63). It has been shown that inhibition of the Ca^2+^/NFAT pathway is involved in the antiproliferative effect of sildenafil on PASMCs (98). More recent studies have revealed that sildenafil inhibits hypoxia-induced TRPC6 protein expression in PASMCs *via* the cGMP–PKG–PPARγ axis (99).

## TRPC6 in Acute Lung Injury (ALI)

Acute lung injury is characterized by lung edema due to increased lung vascular permeability of the alveolar-capillary barrier and subsequent impairment of arterial oxygenation. Ca^2+^ homeostasis has been shown to be essential in the mechanism of barrier disruption and endothelial contraction (3). Elevated [Ca^2+^]_i_ leads to changes in EC morphology and increased endothelial permeability. Recent studies have shown that Ca^2+^ entry through TRPC6 is essential for increased endothelial permeability and compromised barrier function in pulmonary vasculature (100).

In ALI, lung vascular barrier disruption usually coincides with the invasion of immune cells and activation of inflammatory signaling pathways (101). Various mediators, including platelet-activating factor (PAF), vascular endothelial growth factor (VEGF), thrombin, tumor necrosis factor-α (TNF-α), and others, induce changes in EC shape and consequently an increase in endothelial permeability (3, 102). PAF, a critical mediator in numerous experimental models of ALI, has been shown to increase lung vascular permeability by activation of acid sphingomyelinase (ASM) (103). In an extension of that study, the authors provided evidence that ASM activation by PAF causes rapid recruitment of TRPC6 channels into caveolae of lung ECs, thus facilitating endothelial Ca^2+^ entry and subsequent increases in endothelial permeability (104). Translocation of the TRPC6 to caveolin-rich areas in the plasma membrane in response to bradykinin has also been shown to be facilitated by 11,12-epoxyeicosatrienoic acids (105). TRPC6 has also been implicated in the VEGF-mediated increase in [Ca^2+^]_i_ and subsequent downstream signaling in microvascular ECs (106–108). In human pulmonary ECs, interaction of a protein called phosphatase and tensin homolog with TRPC6 enables cell surface expression of the channel in ECs and OAG-induced Ca^2+^ entry through TRPC6 as well as a subsequent increase in monolayer permeability (109). Thrombin-mediated Ca^2+^ entry through TRPC6 in human pulmonary artery ECs activated RhoA in a protein kinase C-α-dependent manner and thereby induced EC shape change and an increase in endothelial permeability (100).

A novel function for TRPC6 in pulmonary ECs in ALI induced by the endotoxin lipopolysaccharide (LPS) has also been indentified (110). In that study, LPS induced generation of DAG by binding to toll-like receptor 4 (TLR4), and DAG in turn directly activated TRPC6 and increased Ca^2+^ entry in ECs resulting in enhanced lung vascular permeability. Most interestingly, TRPC6 signaling was also important for the LPS/TLR4-mediated NF-κB activation and lung inflammation (110).

Lung edema and endothelial injury are accompanied by an influx of neutrophils into the interstitium and alveolar space (111). Therefore, activation and recruitment of polymorphonuclear neutrophils are thought to play key roles in the progression of ALI. When neutrophils are recruited to inflamed tissue, they become migratory and traverse the walls of blood vessels. It is known that the stimulation of CXC-type Gq-protein-coupled chemokine receptors activates PLC and induces a sustained increase in [Ca^2+^]_i_ (112). An important role of TRPC6 signaling was demonstrated in CXCR2-induced intermediary chemotaxis (113). A deficiency for TRPC6 in neutrophil granulocytes negatively affects macrophage inflammatory protein-2 and OAG-induced cell migration (114). It has also been shown that TRPC6 expressed in ECs promotes leukocyte transendothelial migration by mediating trafficking of the lateral border recycling compartment membrane (115).

**Figure 3:**
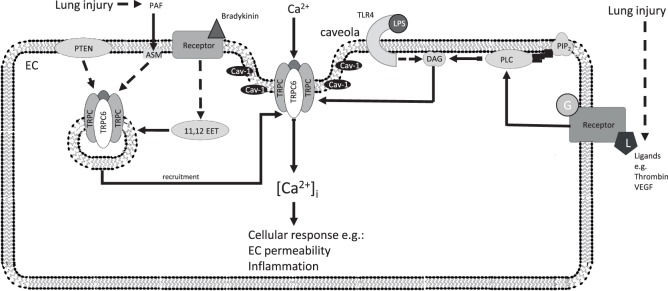
Additional TRPC6 signaling pathways in ECs after lung injury. Recruitment of TRPC6 by the indicated factors increases the density of TRPC6 channels at the plasma membrane (left), which open after activation of endothelial receptors (right) and increase endothelial permeability and inflammatory processes inducing endothelial dysfunction. 11,12 EET, 11,12-epoxyeicosatrienoic acid; ASM, acid sphingomyelinase; Ca^2+^, calcium ion; [Ca^2+^]_i_, intracellular Ca^2+^ concentration; Cav-1, caveolin-1; DAG, diacylglycerol; EC, endothelial cell; G, G-protein; HIF-1α, hypoxia-inducible factor 1 alpha; L, ligand; LPS, lipopolysaccharide; PAF, platelet-activating factor; PTEN, phosphatase and tensin homolog; PIP_2_, phosphatidylinositol 4,5-bisphosphate; PLC, phospholipase C; TLR4, toll-like receptor 4; TRPC, classical transient receptor potential channel; TRPC6, classical transient receptor potential channel 6; VEGF, vascular endothelial growth factor; solid arrows indicate direct interactions; dotted arrows illustrate indirect interactions. Not all interaction partners have been identified.

Recently, we have investigated the role of TRPC6 in lung ischemia-reperfusion edema (LIRE) formation in mice (31). Remarkably, global TRPC6^−/−^ mice were fully protected from LIRE, whereas global TRPC1- and TRPC4-deficient mice showed no protection. Bone marrow transplantation experiments using TRPC6 KO and wild-type mice allowed us to exclude the involvement of TRPC6 in immune cells. In line with our *in vivo* findings, pulmonary ECs isolated from TRPC6 KO mice displayed reduced permeability in response to hypoxia. A detailed analysis of signaling pathways underlying TRPC6 activation showed that mice lacking NOX2, but not NOX1 and NOX4, were also protected from LIRE. Moreover, mice deficient for NOX2 specifically in pulmonary arterial ECs displayed protection from LIRE. Consistent with our *in vivo* findings, we observed enhanced ${\text{O}}_{2}^{-}$ production by endothelial NOX2 during the ischemic (hypoxic) phase. We have shown that after extracellular conversion to hydrogen peroxide (H_2_O_2_), H_2_O_2_ penetrates into the cell, where it inhibits DAGK η_1/2_ activity and activates PLCγ, resulting in DAG accumulation and activation of TRPC6. Furthermore, elevation in [Ca^2+^]_i_ was diminished in ECs lacking either NOX2 or TRPC6, indicating that NOX2 influences TRPC6-dependent Ca^2+^ homeostasis. Our studies provided a unique mechanistic insight into the pathogenesis of LIRE involving production of superoxide by endothelial Nox2, activation of PLCγ, inhibition of DAGK, and DAG-mediated activation of TRPC6 (31). These studies are summarized in Figure 3.

## Concluding Remarks

In summary, TRPC6 channels are involved in various physiological and pathophysiological processes in the pulmonary vasculature. There is a clear evidence for the importance of TRPC6 in the mechanism of acute HPV. Although the role of TRPC6 in chronic hypoxia-induced PH is controversial, there is evolving evidence for an important function of TRPC6 in pulmonary vascular remodeling in IPAH and endothelial barrier disruption in ALI. Therefore, TRPC6 is a promising target for pharmacological interventions. In physiological processes like acute HPV, TRPC6 activators may be useful to redirect blood flow from non-ventilated regions to oxygen-rich regions of the lungs to avoid life-threatening arterial hypoxemia. In pathophysiological processes like excessive vascular remodeling, PH, or enhanced endothelial permeability, inhibitors of TRPC6 channels might represent a valuable approach. Thus, specific drugs designed to target TRPC6 channels have to be identified as a prerequisite to develop new therapeutic strategies in diseases coupled to physiological and pathological functions of TRPC6 channels.

## Author Contributions

MM, AE, CV, and AS drafted the manuscript. MM, AE, CV, HG, RS, TG, AD, NW, and AS revised the manuscript critically for important intellectual content and approved the final version of the manuscript submitted.

## Conflict of Interest Statement

The authors declare that the research was conducted in the absence of any commercial or financial relationships that could be construed as a potential conflict of interest.
